# Bimodal effect of hydrogen peroxide and oxidative events in nitrite-induced rapid root abscission by the water fern *Azolla pinnata*

**DOI:** 10.3389/fpls.2015.00518

**Published:** 2015-07-09

**Authors:** Michael F. Cohen, Sushma Gurung, Giovanni Birarda, Hoi-Ying N. Holman, Hideo Yamasaki

**Affiliations:** ^1^Department of Biology, Sonoma State University, Rohnert Park, CAUSA; ^2^Biological Systems Unit, Okinawa Institute of Science and Technology, OkinawaJapan; ^3^Faculty of Science, University of the Ryukyus, NishiharaJapan; ^4^Center for Environmental Biotechnology, Earth Sciences Division Lawrence Berkeley National Laboratory, Berkeley, CAUSA

**Keywords:** root abscission, apoplast, free radical cleavage, FTIR spectromicroscopy, hydrogen peroxide, nitric oxide, nitrite, plant cell wall loosening

## Abstract

In the genus *Azolla* rapid abscission of roots from floating fronds occurs within minutes in response to a variety of stresses, including exposure to nitrite. We found that hydrogen peroxide, though itself not an inducer of root abscission, modulates nitrite-induced root abscission by *Azolla pinnata* in a dose-dependent manner, with 2 mM H_2_O_2_ significantly diminishing the responsiveness to 2 mM NaNO_2_, and 10 mM H_2_O_2_ slightly enhancing it. Hypoxia, which has been found in other plants to result in autogenic production of H_2_O_2_, dramatically stimulated root abscission of *A. pinnata* in response to nitrite, especially for plants previously cultivated in medium containing 5 mM KNO_3_ compared to plants cultivated under N_2_-fixing conditions without combined nitrogen. Plants, including *Azolla*, produce the small signaling molecule nitric oxide (NO) from nitrite using nitrate reductase. We found *Azolla* plants to display dose-dependent root abscission in response to the NO donor spermine NONOate. Treatment of plants with the thiol-modifying agents *S*-methyl methanethiosulfonate or glutathione inhibited the nitrite-induced root abscission response. Synchrotron radiation-based Fourier transform infrared spectromicroscopy revealed higher levels of carbonylation in the abscission zone of dropped roots, indicative of reaction products of polysaccharides with potent free radical oxidants. We hypothesize that metabolic products of nitrite and NO react with H_2_O_2_ in the apoplast leading to free-radical-mediated cleavage of structural polysaccharides and consequent rapid root abscission.

## Introduction

Small floating water ferns of the genus *Azolla* are model systems for studies of abscission (Cohen et al., 2014), dropping roots within minutes in response to heat (Uheda et al., 1999) and to various chemical exposures, including nitrite (Uheda and Kitoh, 1994; Gurung et al., 2012).

To assess the abscission response intact plants can be placed onto a test solution and observed for root dropping. Alternatively, roots may be pulled from the frond, immersed into test solution and then observed for expansion and separation of abscission zone cells. Studies on pulled roots of *Azolla filiculoides* found that exposure to the protease papain nearly abolished the abscission response, demonstrating an essential function for extracellular proteins (Uheda et al., 1994), and that neither actinomycin D nor cyclohexamide inhibit the abscission response, indicating that such proteins are preformed (Uheda and Kitoh, 1994). An increase in apoplastic pH, which is typically in the range of pH 5–6 in plants (Palmgren, 2001), is likely to be a proximate stimulus for abscission since immersion of pulled roots in buffered solutions having pH ≥ 6.7 results in near immediate cell separation at the abscission zone (Uheda et al., 1994). Consistent with this, immersion in acidic buffers abolishes responsiveness to abscission-inducing compounds (Uheda and Kitoh, 1994) while treatment with various protonophores rapidly induces cell separation (Uheda and Kitoh, 1994).

The finding that the dissolution of middle lamella of abscission zone cells cannot be reproduced by exogenously provided cell wall-degrading enzymes (Uheda et al., 1994; Fukuda et al., 2013), as well as the rapid speed of the process, led to the hypothesis that free radical-mediated breakage of cell wall polysaccharides occurs during rapid abscission (Fukuda et al., 2013). Such oxidative non-hydrolytic cleavage of cell wall polysaccharides has been observed in other processes that involve cell wall loosening, including seed germination, cell elongation (Schopfer, 2001; Müller et al., 2009), and fruit ripening (Fry et al., 2001). Supporting this hypothesis, histochemical evidence of oxidative reactions was found in the abscission zone of abscised roots of *A. filiculoides* and, furthermore, abscission could be induced by treatment of plants with agents that generate hydroxyl radicals (⋅OH) via a Fenton-type reaction (Yamada et al., 2015).

Plant cell wall associated peroxidases are capable of generating ⋅OH that can cleave (Liszkay et al., 2003) and carbonylate cell wall polysaccharides (Fry, 1998; Fry et al., 2001; Marnett et al., 2003). Recent advancements in synchrotron radiation-based Fourier transform infrared (SR-FTIR) spectromicroscopy have allowed for detection of functional groups including carbonyls *in situ* rather than by the traditional analysis of extracts (Holman et al., 2010; Lacayo et al., 2010). One limitation in applying this technique is that samples must be thin enough to allow for transmission of the light beam. In this regard the thinness of *Azolla* roots presents an advantage since there is no need to carry out thin-sectioning of samples prior to spectroscopic analysis.

Here we report pharmacological studies that indicate a role for thiol-targeted oxidative events in regulating the abscission process as well as SR-FTIR spectromicroscopic evidence of free radical attack in the abscission zone of dropped roots.

## Materials and Methods

### Plant Material and Surface Sterilization

Laboratory cultures of *Azolla pinnata* were established from plants collected in December 2012 and May 2014 from a taro field in Ginowan, Okinawa, Japan. The plants were thoroughly washed to remove attached mud and debris. The plants were then treated with a solution of 0.12% sodium hypochlorite and 0.01% Triton X-100 for 30 min followed by repeated washings in a large volume of distilled water and finally transferred into nutrient medium (Peters and Mayne, 1974).

### Nutrient medium and culture conditions

*A. pinnata* was cultured in a two-fifth strength cobalt-supplemented nitrogen-source-free Hoagland’s E-medium (Gurung et al., 2012). Medium pH was adjusted to 5.8 with potassium hydroxide. Plants were grown in a plant growth chamber (Type FLI-2000H, Eyla, Japan) maintained at 27 ± 1°C, 80% humidity, 16:8 h light:dark photoperiod and 50 μmol m^-2^ s^-1^ (at plant level) provided by fluorescent lamps (Type FL 40 SBR-A, NEC, Japan). For experiments 15 to 20 fronds were randomly selected from the culture stock and de-rooted manually using forceps. Rootless fronds were cultured in nutrient medium after rinsing in distilled water. The plants were transferred to a fresh medium every 4 days.

### Synchrotron Radiation-Based Fourier Transform Infrared Spectromicroscopy

Plant samples were prepared for SR-FTIR spectromicroscopy by freeze drying. Roots from *A. pinnata* were selected under a visible microscope and enclosed between two CaF_2_ infrared crystals in order to assure the planarity of the sample. For each treatment at least two regions were selected for measurement, one close to the abscission zone and one few millimeters apart, as an internal control. IR maps were collected at infrared beamlines of the Advanced Light Source (http://infrared.als.lbl.gov/), mid-infrared photons (∼2.5–15.5 μm wavelength λ, or ∼4000–650 cm^-1^ wavenumber) emitted from the synchrotron bending magnet are focused down to a 0.76λ diffraction-limit spot size with a Nicolet Continuum infrared microscope, coupled to a Nicolet 6700 FTIR Spectrometer (Thermo Fisher Scientific Inc.), using a 32X objective with 0.65 numerical aperture. Each map was acquired with a spatial resolution of 5 μm. Spectra were corrected for water vapor and CO_2_ and converted in ENVI format. An average spectrum was calculated for abscission and control zones from all maps and treatments, and then a cluster analysis was performed calculating the Euclidian distances of the second derivative of vector normalized spectra in the range from 1800 to 1200 cm^-1^ using the Ward’s method for the agglomerative hierarchical clustering procedure. Wavenumber FTIR peak identities were assigned based on Socrates (2004). Moreover seasonal differences were identified by analyzing the spectral difference of the controls. The same approach was used to highlight chemical differences in the samples due to the specific treatment, considering the difference between treated and control from the same batch, and chemical differences of the abscission zone from the control zone in the same root. The difference spectra were calculated after applying vector normalization on the whole 1800–1200 cm^-1^ interval.

### Chemical Treatment of *A. pinnata*

Abscission assays were carried out using roots of equal age (i.e., from fronds that had been de-rooted at the same time, 9–12 days prior to preforming the assay). 2 to 3 fronds (20–30 roots) were suspended in a beaker containing 20 ml 10 mM potassium phosphate buffer at pH 7. The chemicals to be tested, sodium nitrite (hereafter referred to as nitrite), H_2_O_2_, 2-(4-carboxyphenyl)-4,4,5,5-tetramethyl-imidazole-1-oxyl-3-oxide (carboxy-PTIO), and spermine NONOate (SNN; Cayman Chemicals Company, Ann Arbor, MI, USA) were subsequently supplied as concentrated stock solutions. The total number of dropped roots following addition of the chemicals was recorded every 10 min for up to 2.5 h. The abscission response was quantified as the ratio of the detached to the initial number of roots.

For some trials the plants were co-incubated or pre-incubated with other chemicals in 10 mM potassium phosphate buffer. 20 mM glutathione (GSH) was added to 2 mM nitrite-treated plants at three time intervals: at the same time (*t* = 0) and 30 and 60 min after initiation of nitrite treatment. Nitrite-treated plants without any GSH addition served as controls. For trials testing the effect of *S*-methyl methanethiosulfonate (MMTS), which covalently sulfenylates the sulfur of cysteine residues, plants were pre-incubated with 1 mM MMTS for 60 min followed by addition of 4 or 6 mM nitrite. We used higher levels of nitrite for this set of experiments because this batch plants showed less than typical responsiveness to 2 mM nitrite.

### Statistics

The means of abscission responses to treatments, quantified as percent of roots dropped, were compared by ANOVA and subjected to a Tukey-Kramer test of significance using SAS/STAT(R) 9.22 (SAS Institute Inc., Cary, NC, USA).

## Results

### Combinatorial Effect of Nitrite and Hydrogen Peroxide on *A. pinnata* Root Abscission

**FIGURE 1:**
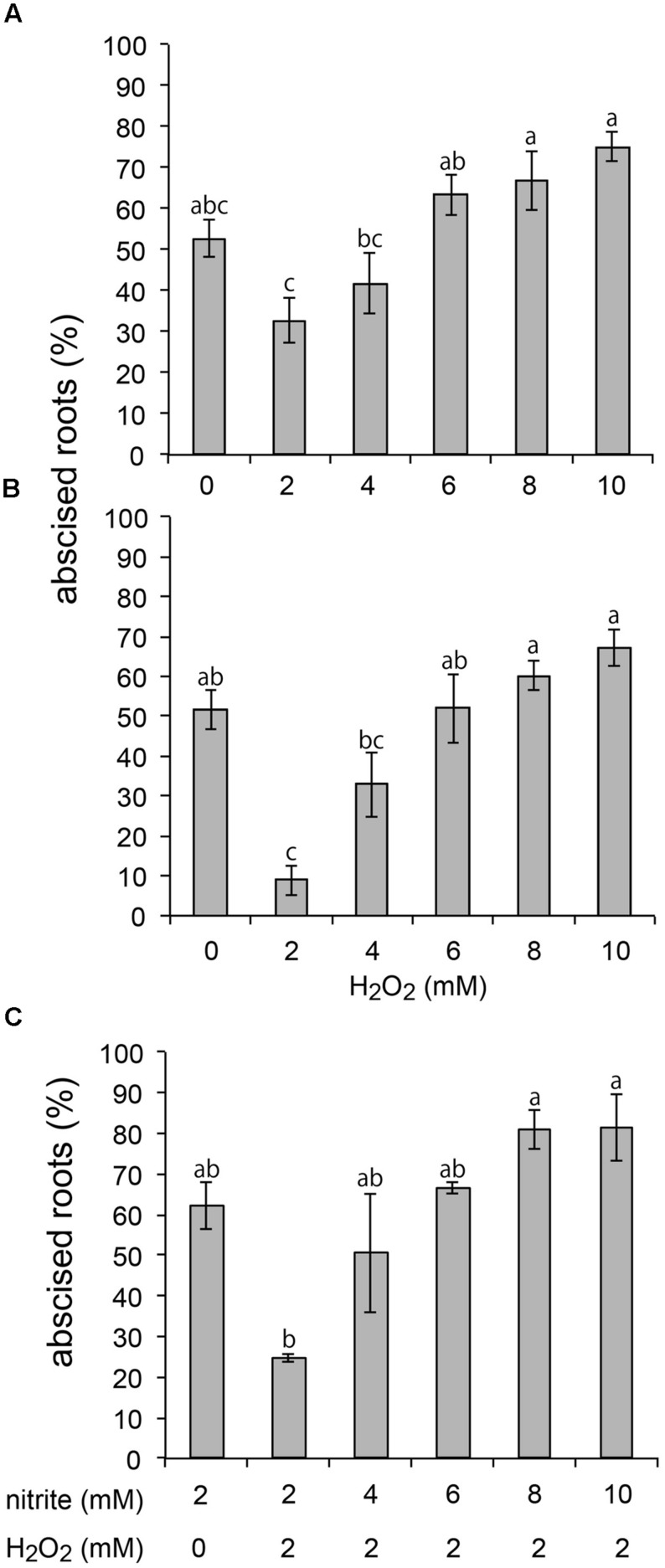
**Combined effect of nitrite and H_2_O_2_ on root abscission of *Azolla pinnata* plants.** Abscission was measured 90 min after 2 mM nitrite was added. 2, 4, 6, 8, or 10 mM H_2_O_2_ were added **(A)** simultaneous with or **(B)** 60 min before addition of nitrite. **(C)** 2 mM H_2_O_2_ was added simultaneously with 2, 4, 6, 8, or 10 mM nitrite (**A**, *n* = 7; **B**, *n* = 8, **C**, *n* = 2, mean ± SE; means with the same letter are not significantly different).

In other plant systems Sakamoto et al. (2008) have described a H_2_O_2_ burst coinciding with leaf abscission that would presumably provide substrate for ⋅OH generation (Liszkay et al., 2003). This H_2_O_2_ burst is apparently distinct from the lower level of H_2_O_2_ production that functions in the abscission signaling process. We have previously reported that treatment of *A. pinnata* plants with up to 10 mM H_2_O_2_ alone does not induce root abscission (Gurung et al., 2012). However, in combination with the known abscission-inducing agent nitrite, treatment of plants with H_2_O_2_ exhibited a pronounced effect that depended on the concentration and timing of the H_2_O_2_ treatment relative to exposure to nitrite (**Figures 1A,B**). The inhibitory effect of 2 mM H_2_O_2_ on root abscission induced by 2 mM nitrite could be overridden by the further addition of nitrite (>4 mM nitrite; **Figure 1C**).

**FIGURE 2:**
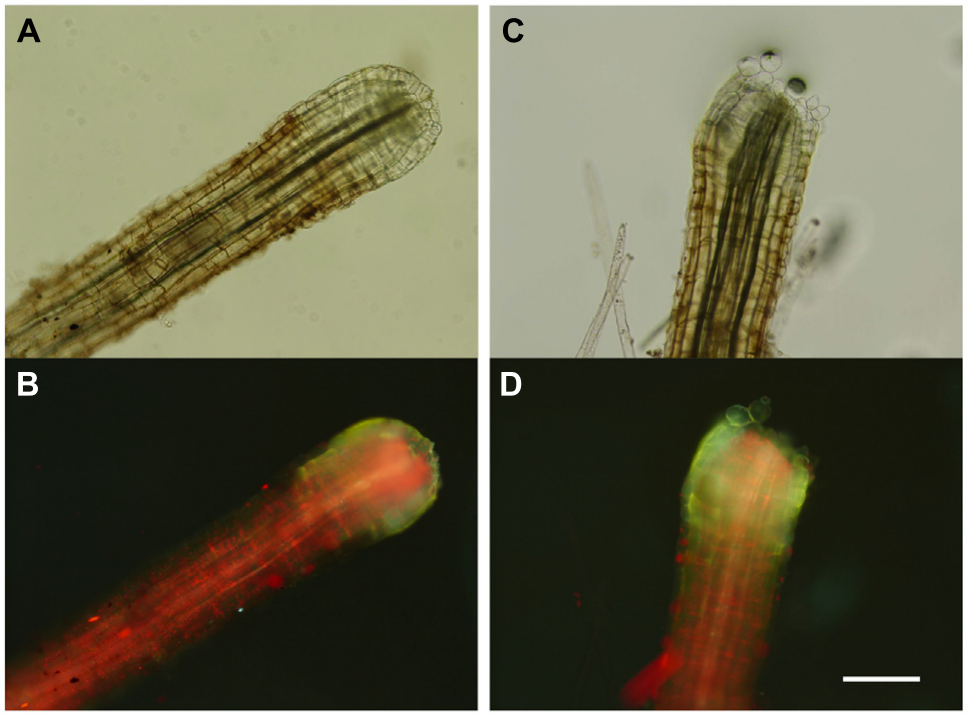
**Effect of combined treatment with nitrite and hydrogen peroxide on proximal root ends.** Representative proximal ends of *A. pinnata* roots from plants were **(A,B)** untreated and removed from the frond by pulling or **(C,D)** dropped following simultaneous treatment with 2 mM nitrite and 10 mM H_2_O_2_ for 120 min. **(A,C)** Light, **(B,D)** fluorescence (excitation 380 nm) microscopic images; scale bar, 200 μm.

Fluorescence, indicative of phenolics accumulation (Yamasaki et al., 1995), in the root tip region formerly inserted in the frond was not discernibly different in roots dropped from plants treated with 2 mM nitrite and 10 mM H_2_O_2_ compared to the same region of untreated pulled roots (**Figure 2**). Release of bubbles following addition of H_2_O_2_ (presumably due to catalase activity) was qualitatively stronger at the root tip than other portions of the root and was noticeably diminished in roots that had been abscised by following exposure to 5 mM nitrite (Supplementary Figure S1).

**FIGURE 3:**
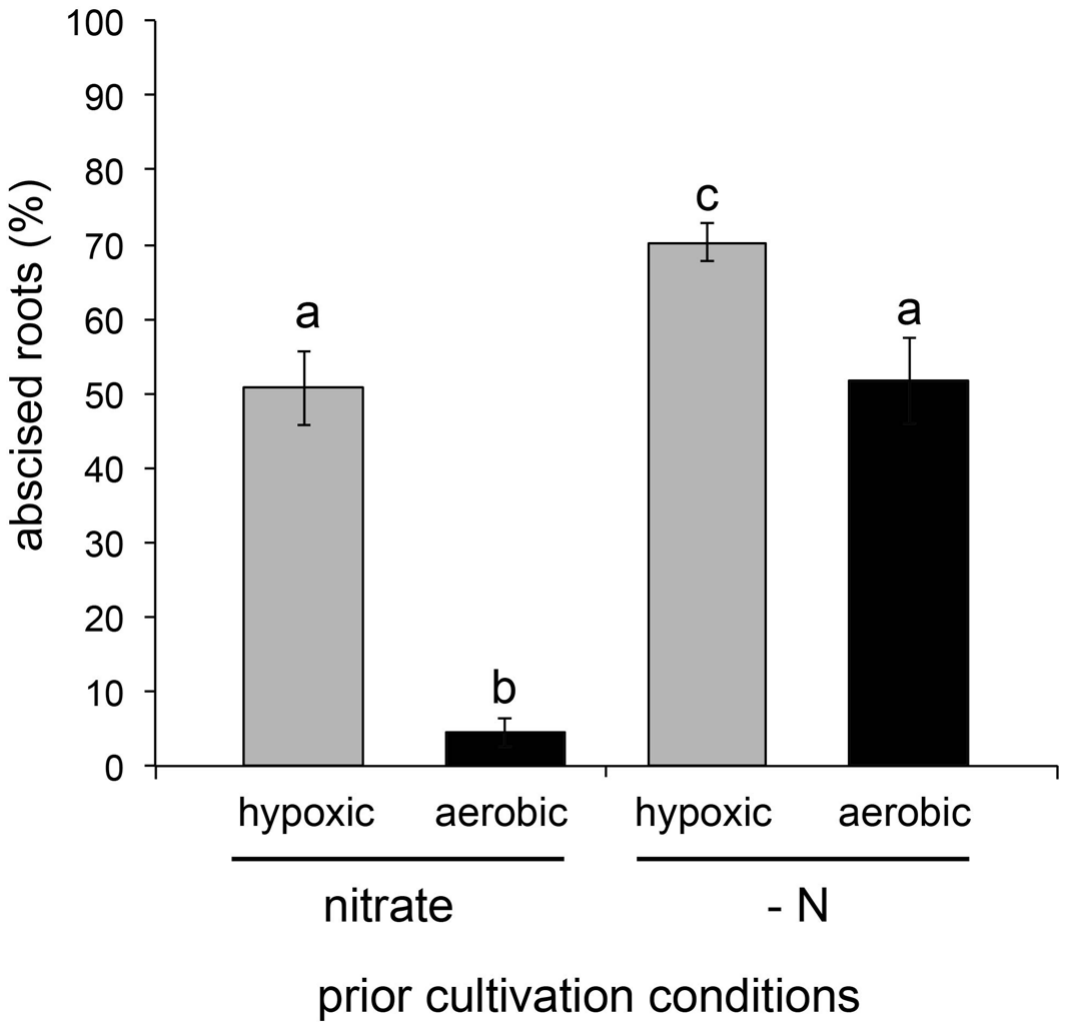
**Effect of hypoxia on abscission induced by 2 mM nitrite treatment of plants with or without prior cultivation in medium containing 5 mM nitrate.** Abscission under hypoxic or aerobic conditions was measured 150 min after addition of nitrite (means ± SE; hypoxia, *n* = 4; aerobic, *n* = 6).

Exposure of plants to hypoxia induces generation of H_2_O_2_ (Blokhina et al., 2001). We observed that hypoxia in combination with 2 mM nitrite induced abscission to a greater extent than did treatment with 2 mM nitrite alone (**Figure 3**); hypoxia without nitrite supplementation did not induce abscission (results not shown). Plants that had been cultivated in medium containing 5 mM potassium nitrate prior to the assay showed significantly less responsiveness to hypoxia (**Figure 3**).

**FIGURE 4:**
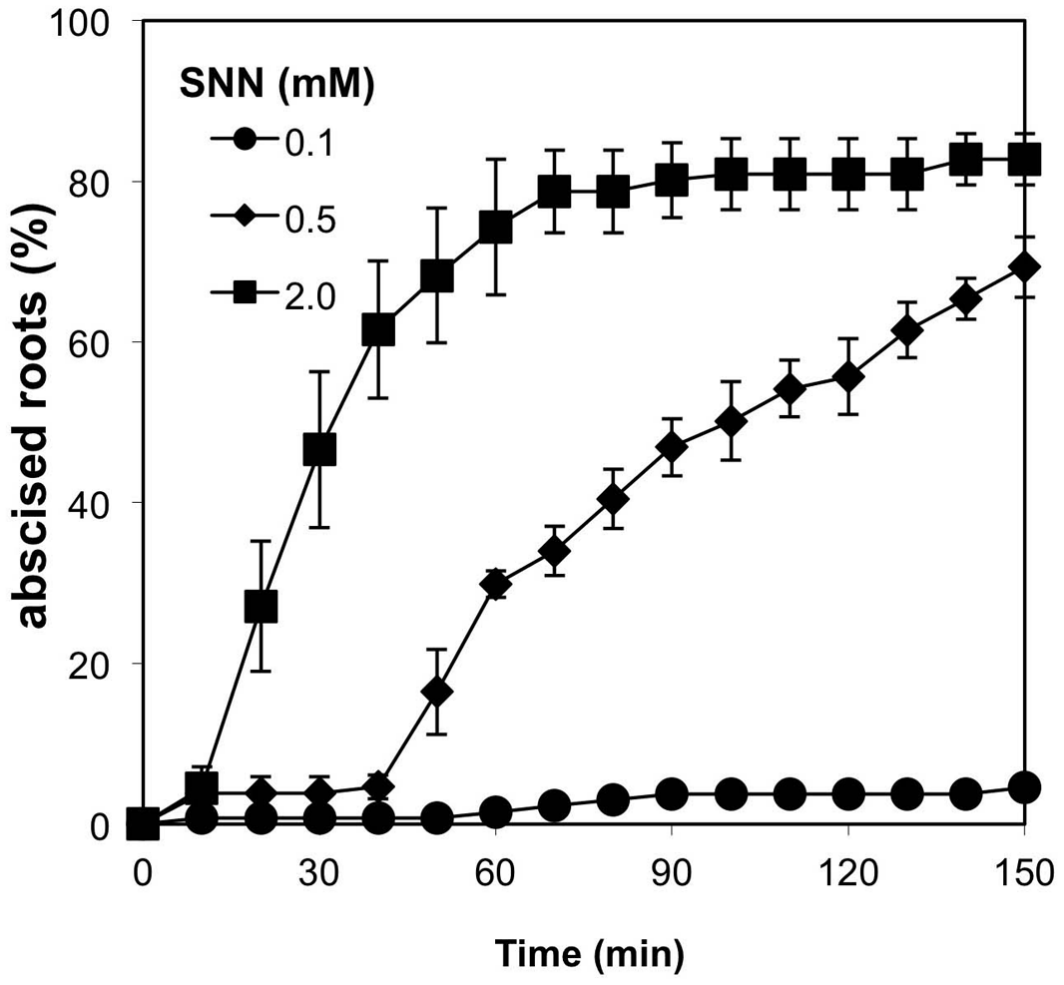
**Dose-dependent induction of *A. pinnata* root abscission by spermine NONOate (SNN).** 0.1 mM (*n* = 3), 0.5 mM (*n* = 4), 2.0 mM (*n* = 4; means ± SE).

Plants, including *A. pinnata*, can convert nitrite to nitric oxide (NO; Yamasaki, 2005; Gurung et al., 2012), from which a variety of reactive nitrogen species (RNS) can be generated (Fukuto et al., 2012). A role for NO in nitrite-induced root abscission was implied by the reduction in abscission observed of nitrite-treated plants in the presence carboxy-PTIO, which oxidizes NO; the inhibitory effect of carboxy-PTIO was substantially diminished by increasing the concentration of nitrite (Supplementary Figure S2). Consistent with this finding, treatment of plants with the NO donor SNN elicited a strong dose-dependent abscission induction response (**Figure 4**).

### Thiol-Active Treatments Suppressing Induction of Abscission

**FIGURE 5:**
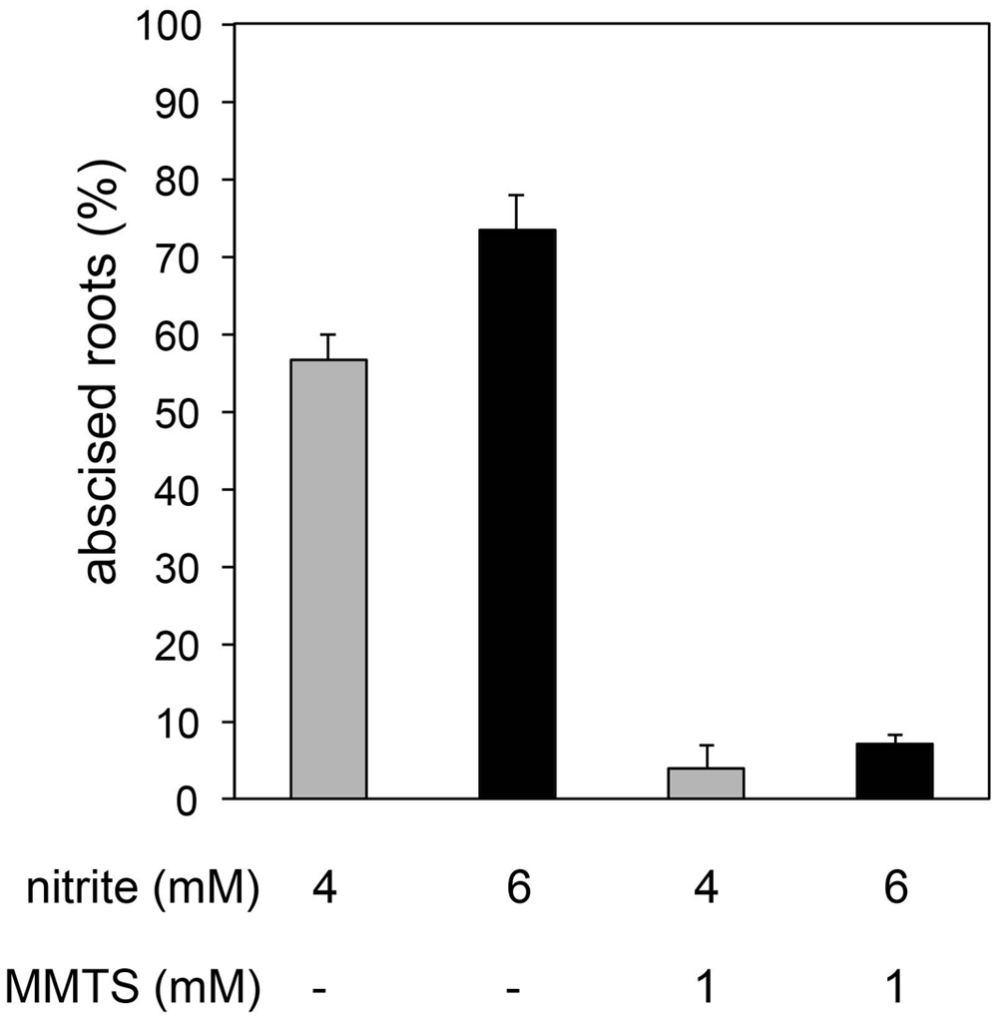
**Effect of the thiol-sulfenylating agent methyl methanethiosulfonate (MMTS) on nitrite-induced root abscission.** Plants were pre-treated with 1 mM MMTS for 60 min and abscission measured 90 min after addition of nitrite (means ± SE; 4 mM nitrite *n* = 5, 6 mM nitrite, *n* = 3).

**FIGURE 6:**
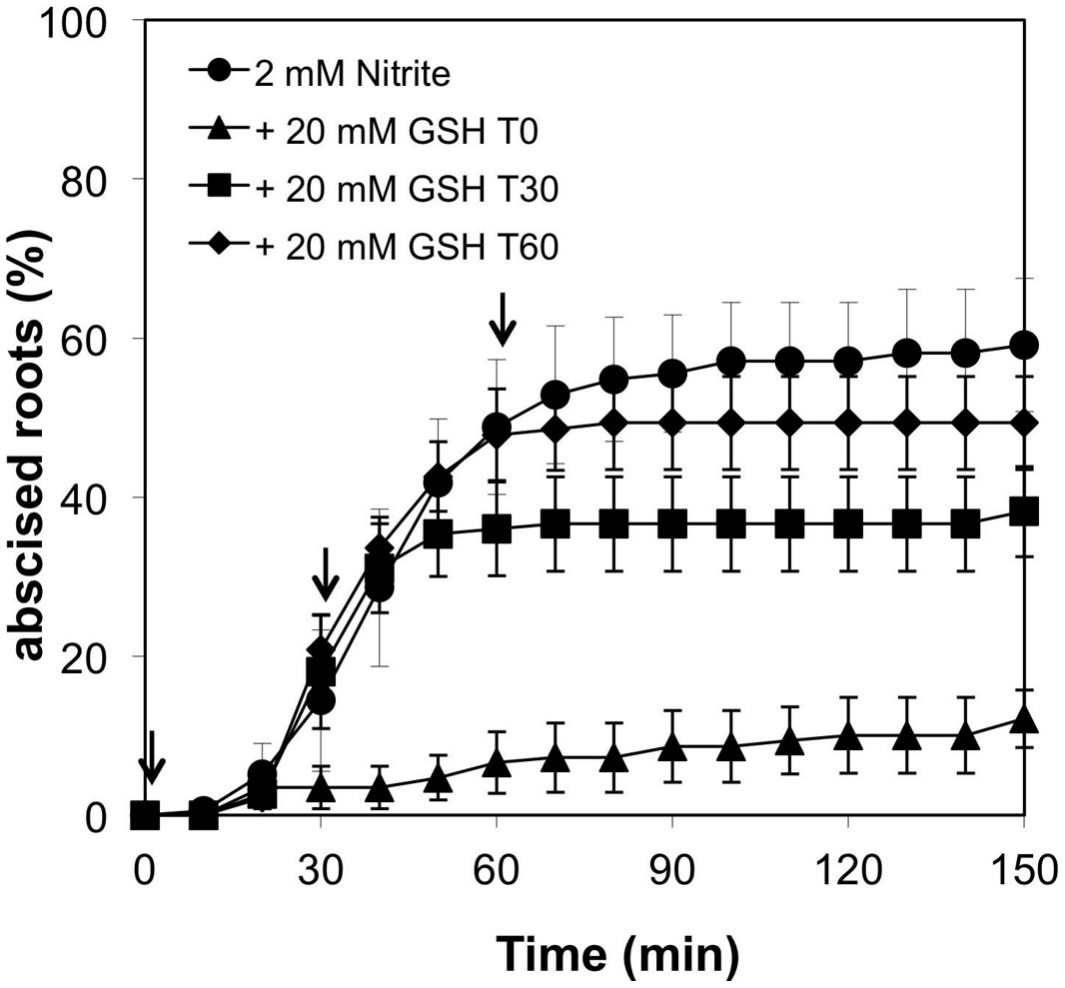
**Effect of glutathione (GSH) on nitrite-induced root abscission.** 20 mM GSH was added at various time points (arrows) following addition of 2 mM nitrite (means ± SE, *n* = 4).

Nitrite-induced abscission was suppressed by treatment of plants with MMTS (**Figure 5**) or GSH (**Figure 6**). 20 mM GSH nearly prevented the abscission response to 2 mM nitrite and when added at 30 or 60 min following nitrite prevented further abscission (**Figure 6**).

### SR-FTIR Demonstration of Abscission Zone Carbonyl Formation

**FIGURE 7:**
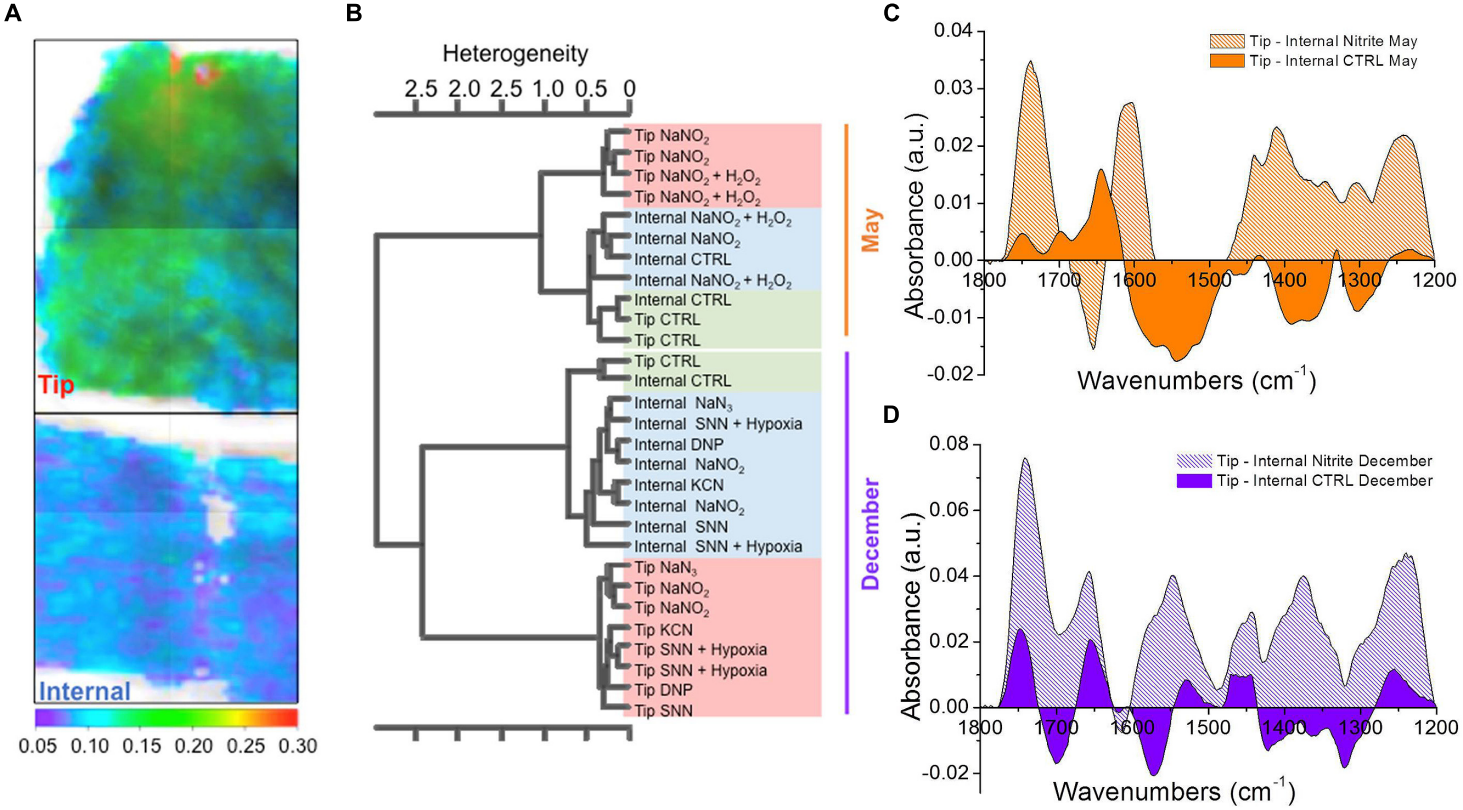
**Synchrotron radiation-based Fourier transform infrared (SR-FTIR) spectromicroscopy of *A. pinnata* roots. (A)** Micrograph showing representative SR-FTIR-scanned regions of a root tip and internal part of an abscised root overlain with the respective chemical maps of ratio C=O (non-peptide) signal (1780–1705 cm^-1^) to the C–O–R signal (1705–1550 cm^-1^). **(B)** Cluster analysis of SR-FTIR spectra taken of regions at abscised root tips, internal regions >1 mm away from abscised root tips, and at the abscission zone and internal regions of untreated pulled roots. Chemical treatments were 2 mM NaNO_2_, 10 mM H_2_O_2_, 1 mM SNN and known inducers of root abscission 0.5 mM NaN_3_, 1 mM dinitrophenol (DNP), and 5 mM KCN (Uheda and Kitoh, 1994). **(C,D)** Representative SR-FTIR difference spectra of tip and internal root regions of NaNO_2_-treated and untreated plants sampled in May **(C)** and December **(D)**.

The chemical maps presented in **Figure 7A** show the SR-FTIR intensity of the ratio of the C=O (non-peptide) signal (1780–1705 cm^-1^) to the C–O–R signal (1705–1550 cm^-1^); the ratio was chosen in order to not be affected by difference of thickness between the measured areas. The tips encompassing the abscission zone of dropped roots are richer in C=O than other root parts. By using agglomerative cluster analysis on the average spectra of *A. pinnata* exposed to different conditions, we can observe that there always is a segregation of tips (red in **Figure 7B**) and internal parts (blue in **Figure 7B**) of the roots. This phenomenon means that the spectral features, hence chemical composition, of the tips are more similar between themselves than the other internal parts of the same roots and the tips of the untreated control roots (green in **Figure 7B**). Looking at the difference spectra presented in **Figures 7C,D** it can be seen that the abscised tips show increases in signal at ∼1740 cm^-1^ in the SR-FTIR spectrum, indicative of carbonyl groups that are formed upon ⋅OH attack of cell wall polysaccharides (Fry, 1998; Fry et al., 2001; Marnett et al., 2003). This increase occurs on top of a pre-existing peak in this range arising from C=O containing molecules in the plant tissue, including carboxylic acid groups from methylated pectin (Szymanska-Chargot and Zdunek, 2013). Importantly, in the frond attachment point of untreated roots this characteristic peak is from 4 to 6 times less intense. Appearance of carbonyl groups would not be expected if only enzymatic hydrolysis of polymers were occurring in the cell wall. Thus, these results support the model for ⋅OH-mediated cell wall dissolution. Furthermore, evidence of increased oxidation in the treatment can be seen in peaks within the range 1430–1335 cm^-1^ due to the symmetric stretching of CO_2_^-^ in carboxylates and 1320–1210 cm^-1^ from the stretching of C-O groups, which can result from ⋅OH attack of biological molecules (Nappi and Vass, 1998). Lastly, we can assign the signal at 1460–1430 cm^-1^ to peroxy acids, whose O–H bending shifts toward 1430 cm^-1^ for long-chain linear acids that may originate from the oxidation of cell wall carbohydrates. Other important signals that are clearly detectable in the difference spectra of the nitrite treatments in **Figures 7C,D** are those arising from NO_3_^-^ and NO_2_^-^, respectively, at 1380 and 1254 cm^-1^ (Mallard, 2015).

Seasonal differences are apparent in **Figure 7B**, which shows primary clustering by sampling date (December and May), and in comparing the distinct difference spectra of tips of untreated roots in **Figures 7C,D**. Large-scale seasonal changes in tissue concentrations deoxyanthocyanins, carotenoids, and chlorophyll known to occur in *Azolla* species (Cohen et al., 2002; Kösesakal, 2014) may account for these seasonal spectral differences.

## Discussion

Previous reports have pointed to the functioning of preformed extracellular proteins in the rapid root abscission process of *Azolla* (Uheda and Kitoh, 1994) with evidence of free radical, rather than hydrolytic enzyme, mediated cleavage of apoplastic polysaccharides (Fukuda et al., 2013; Yamada et al., 2015). Our results show the abscission process to be influenced by agents that react with or generate compounds that react with thiols, including GSH, MMTS, and H_2_O_2_ in combination with nitrite (Heinecke and Ford, 2010). The effect of H_2_O_2_ on nitrite-induced abscission, from inhibitory at <4 mM to stimulatory at >8 mM, is consistent with the bimodal action of H_2_O_2_ on leaf abscission in other systems, behaving as a regulator at low concentrations and as an agent of cell wall dissolution at higher concentrations (Nappi and Vass, 1998). The greater inhibition of nitrite-induced abscission following 60 min pretreatment with H_2_O_2_ (**Figure 1B**) compared to concomitant addition (**Figure 1A**) could imply H_2_O_2_ acting as a signal to activate the antioxidant capacity, including upregulating biosynthesis of thiol compounds like GSH, as has been shown in other plant systems (Foyer and Noctor, 2011). Higher concentrations of H_2_O_2_ may favor conditions that generate ⋅OH-like potent oxidants with consequent cell wall loosening events (Supplementary Figure S3; Cohen et al., 2014 and references therein).

The positive effect of hypoxia on nitrite-induced abscission may be due to hypoxia-stimulated production of H_2_O_2_; transition into hypoxia is known to result in H_2_O_2_ production in plants (Blokhina et al., 2001) but was not measured in our experiments. The lower responsiveness of nitrate-grown plants to nitrite may be due to competition of nitrate with nitrite for the active site of nitrate reductase, thereby lowering the production of NO by this enzyme (Rockel et al., 2002). Consistent with this observation, tungsten, a non-specific inhibitor of nitrate reductase, also significantly lowered abscission responsiveness to nitrite (Gurung, 2014).

The putative thiol in the target protein may be positioned such that it can only be modified via reactions that occur at the nearby iron rather than through external nitrating agents like NO_2_ gas. The finding that, of the many RNS molecules and donors tested, only the polyamine NO donor SNN induced abscission may indicate a necessity for generating near the site of action; the affinity of polyamines for plant cell walls is well documented (Zepeda-Jazo et al., 2011). Spermine can itself serve as an inducer of root abscission but the response does not initiate until after 50 min of exposure and does not require the activity of the H_2_O_2_-producing polyamine oxidase (Gurung et al., 2012). NO may also be involved in the abscission-inducing mechanism of the most potent inducer of *Azolla* root abscission, sodium azide (Uheda and Kitoh, 1994), which is converted to NO (presumably via HNO) by catalase (Gurung et al., 2014).

The presumptive apoplastic protein target of chemical abscission inducers is most likely already positioned in the apoplast before the abscission-inducing event, based on the rapidity of the response and its insensitivity to cycloheximide (Uheda and Kitoh, 1994). In citrus, Gaspar et al. (1978) demonstrated that activity of preformed peroxidases are upregulated in abscission zones in response to ethylene treatment. Cell wall peroxidases are capable of catalyzing the production of ⋅OH from H_2_O_2_ (Liszkay et al., 2003) and could conceivably be a regulatory target in *Azolla* abscission zones (Cohen et al., 2014). Several lines of evidence implicate free radical attack in the dissolution of pectin seen during abscission in *Azolla* and other plants (Fry et al., 2002; Fukuda et al., 2013; Yamada et al., 2015) and are consistent with the SR-FTIR spectromicroscopic results reported here.

An increase in the FTIR signal at ∼1740 cm^-1^ as we have observed in the *Azolla* root abscission zone has been observed of other oxidative plant cell wall degradation processes that are known to result in formation of C=O groups, including UV-irradiation (Müller et al., 2003) and microbial degradation of wood (Gilardi et al., 1995) and periodate oxidation of cellulose (Spedding, 1960).

Our findings provide more evidence for ⋅OH formation in cell wall cleavage events during the abscission process of *A. pinnata* and lay a foundation for use of SR-FTIR spectromicroscopy for abscission studies. The demonstration of this application for SR-FTIR microscopy is important because tools for investigating the role of hydroxyl radicals in the abscission process are currently limited to ^3^H fingerprinting (Müller et al., 2009). In spite of the rapidity of their abscission response and ease of experimental manipulation *Azolla* species have been considered to be “not suitable for biochemical studies on abscission because the abscission zone is very small” (Uheda and Nakamura, 2000). With the advent of SR-FTIR microscopy the small size of *Azolla* roots can now be seen as an advantage for study since their thinness allows for the penetration of the light beam, allowing us to examine biochemical changes more readily in *Azolla* than in the thicker abscission zones of other species.

## Conflict of Interest Statement

The authors declare that the research was conducted in the absence of any commercial or financial relationships that could be construed as a potential conflict of interest.
