# Supplementary material for: Bimodal effect of hydrogen peroxide and oxidative events in nitrite-induced rapid root abscission by the water fern Azolla pinnata
Source: Front Plant Sci. 2015 Jul 9;6:518. doi: 10.3389/fpls.2015.00518 (PMC4496558; doi:10.3389/fpls.2015.00518)
Supplement: Supplementary file 1 [file Image_1.PDF]

frontiers

Supplementary Material

Bimodal effect of hydrogen peroxide and oxidative events in nitrite-induced rapid root abscission by the water fern *Azolla pinnata*

Michael F. Cohen[1,2*], Sushma Gurung[3], Giovanni Birarda[4], Hoi-Ying N. Holman[4], Hideo Yamasaki[3]

[1]Department of Biology, Sonoma State University, Rohnert Park, CA, USA
[2]Biological Systems Unit, Okinawa Institute of Science and Technology, 1919-1 Tancha, Onna-son, Okinawa, 904-045, Japan
[3]Faculty of Science, University of the Ryukyus, Nishihara 903-0213, Japan
[4]Center for Environmental Biotechnology, Earth Sciences Division, Lawrence Berkeley National Laboratory, Berkeley, CA, USA

*** Correspondence:** Corresponding Author, Department of Biology, Sonoma State University, Rohnert Park, CA, USA.
cohenm@sonoma.edu

1. Supplementary Material

Nitrite inhibition of root catalase activity. Qualitative evidence of catalase activity as bubbling from roots that had been pulled from untreated plants was compared to that of roots that had abscised from plants treated with 5 mM $NaNO_2$. The untreated pulled roots exhibited much more intensive bubbling at the abscission zone than did the abscised roots and showed bubbling at other locations along the root while the abscised root did not (Supplementary Figure 1). The diminished level bubbling in the abscised root is presumably due to residual inhibition of catalase by nitrite (Clark et al., 2000). From these results we cannot distinguish whether the increased bubbling observed at the abscission zone is due to higher production at that site or whether the gas is simply moving through the vascular system and exiting through the path of least resistance.

Minimal abscission following exposure to NO_x gasses. As seen in Supplementary Figure 2 low level of abscission occurred in response to exposure to 350 ppb NO and while 133 ppm NO and 133 ppm NO_2 did not induce abscission.

A hypothetical scheme. Supplementary Figure 3 depicts a hypothetical model for H_2O_2 and NO in apoplastic thiol regulation of free radical-mediated abscission zone cell expansion events.

1.1. Supplementary Figures

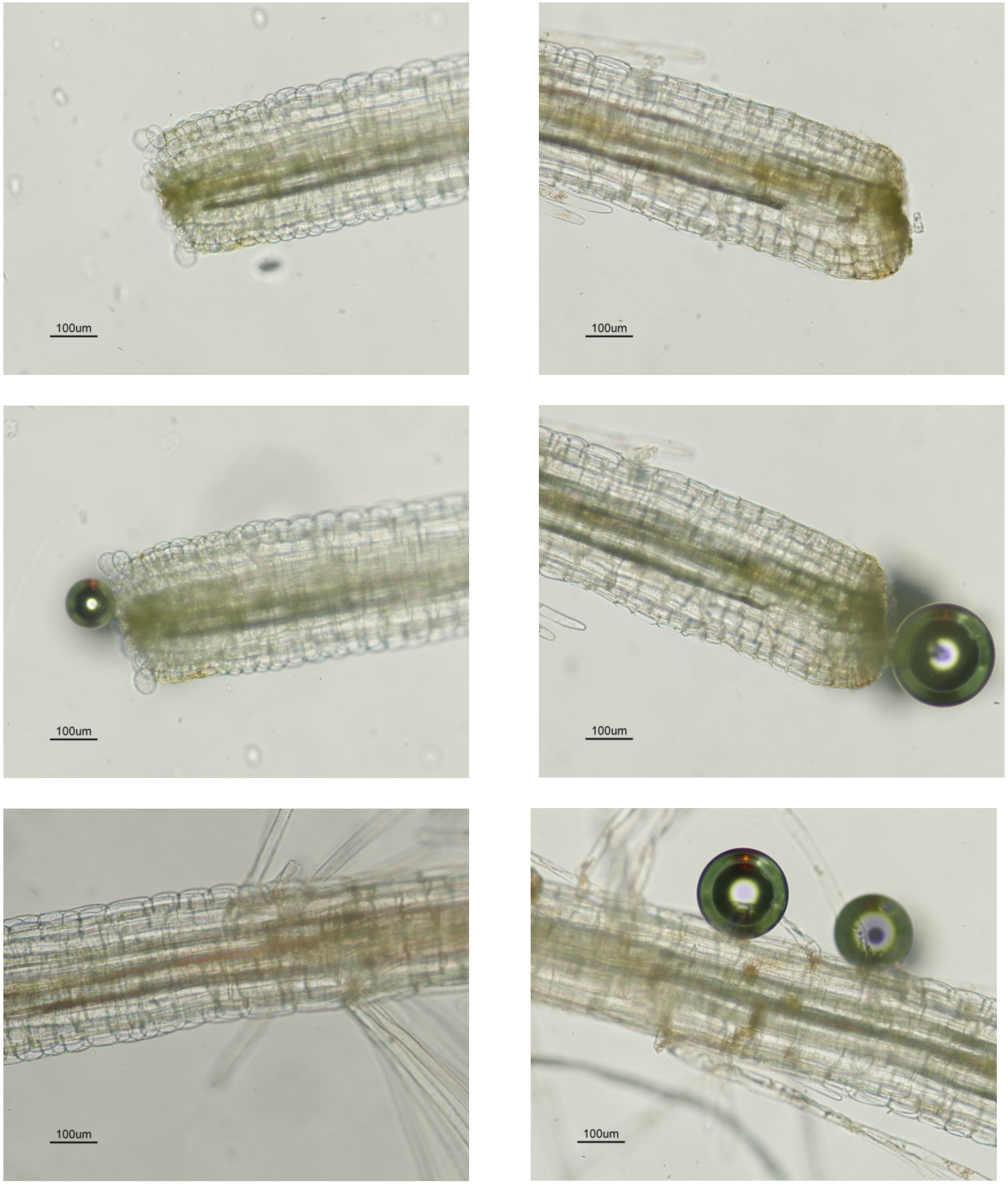

Supplementary Figure 1. Catalase activity by roots of *Azolla pinnata* treated with 16.7 mM H_2O_2 and its inhibition by nitrite. *Left*, dropped root from a plant treated for 30 min with 5 mM $NaNO_2$. *Right*, pulled root from a plant without nitrite pretreatment showing bubble formation indicative of catalase activity. *Top*, proximal tips of the roots immediately following addition of H_2O_2. *Middle*, proximal root tips 15 min following addition of H_2O_2. *Bottom*, representative internal root locations 20 min following addition of H_2O_2.

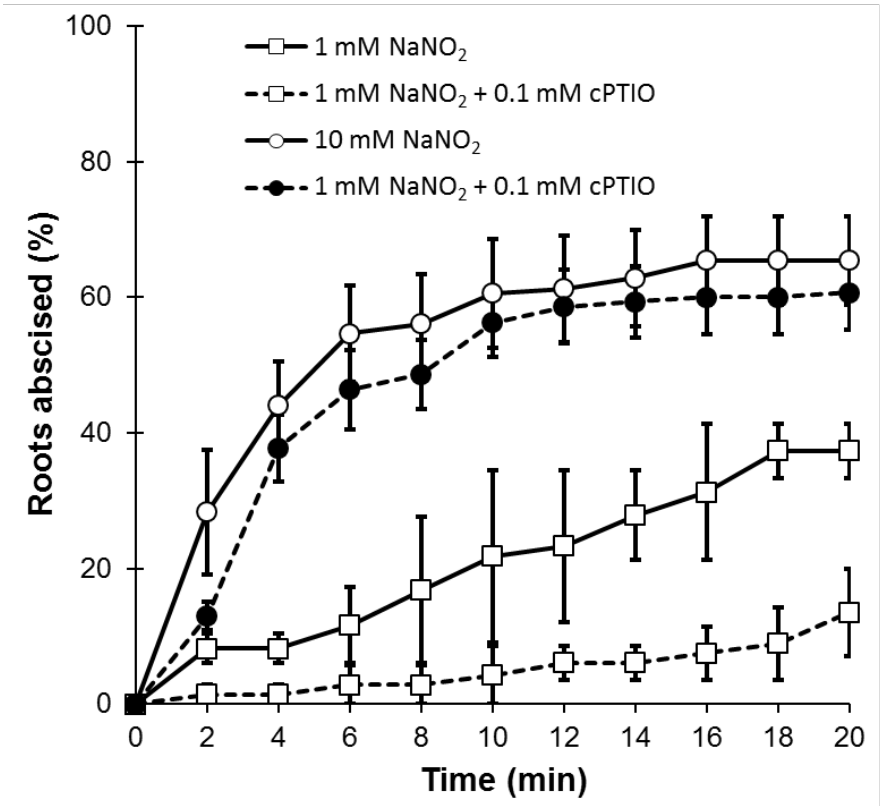

Supplementary Figure 2. Effect of treatment with the NO scavenger 2-(4-carboxyphenyl)-4,4,5,5-tetramethyl-imidazole-1-oxyl-3-oxide (cPTIO) on nitrite-induced abscission of *Azolla pinnata* plants (means ± SE, n = 2 for 1 mM $NaNO_2$ treatments, n = 5 for 10 mM $NaNO_2$ treatments).

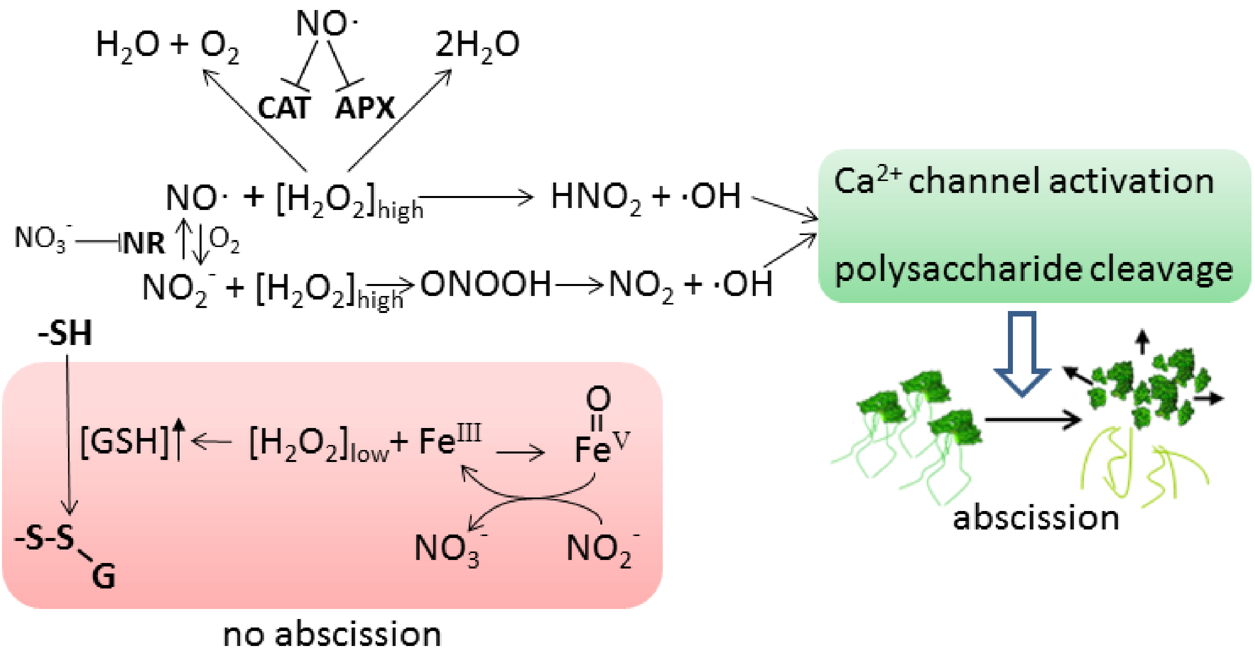

Supplementary Figure 3. A scheme depicting hypothetical roles for putative apoplastic thiols and free-radical generating reactions in nitrite-induced cell expansion of *Azolla* abscission zone cells. H_2O_2 diminishes sensitivity to nitrite at low H_2O_2 concentrations but slightly increases sensitivity at high concentrations (Fig. 1). At low H_2O_2 concentrations glutathione (GSH) biosynthesis would be upregulated (Foyer and Noctor, 2011) and iron would catalyze H_2O_2-mediated oxidation of nitrite to nitrate, which inhibits production of NO· by nitrate reductase (NR). As iron becomes saturated at higher concentrations of H_2O_2 the reactions between H_2O_2 and nitrite and nitrite-derived NO· would generate potent ·OH-like oxidants (Nappi and Vass, 1998). Production of NO· would prevent H_2O_2 consumption by inhibiting catalase (CAT) and ascorbate peroxidase (APX) (Correa-Aragunde et al., 2015; Clark et al., 2000). Increased ·OH production in the apoplast could result in cell wall expansion both by activating Ca^{2+} channels (Mori and Schroeder, 2004) and cleaving cell wall polysaccharides (Cohen et al., 2014). Yamada et al. (2015) were able to induce expansion of abscission zone cells by exposing in detached roots of *Azolla filiculoides* to Fenton reaction-generated ·OH.

1.2 Supplementary References

Correa-Aragunde, N., Foresi, N., and Lamattina, L. (2015). Nitric oxide is a ubiquitous signal for maintaining redox balance in plant cells: regulation of ascorbate peroxidase as a case study. *Journal of Experimental Botany* 66**,** 2913-2921.

Clark, D., Durner, J., Navarre, D.A., and Klessig, D.F. (2000). Nitric oxide inhibition of tobacco catalase and ascorbate peroxidase. *Molecular Plant-Microbe Interactions* 13**,** 1380-1384.

Cohen, M.F., Gurung, S., Fukuto, J.M., and Yamasaki, H. (2014). Controlled free radical attack in the apoplast: A hypothesis for roles of O, N and S species in regulatory and polysaccharide cleavage events during rapid abscission by Azolla. *Plant Science* 217**,** 120-126.

Mori, I.C., and Schroeder, J.I. (2004). Reactive oxygen species activation of plant Ca^{2+} channels. A signaling mechanism in polar growth, hormone transduction, stress signaling, and hypothetically mechanotransduction. *Plant Physiology* 135**,** 702-708.

Nappi, A.J., and Vass, E. (1998). Hydroxyl radical formation resulting from the interaction of nitric oxide and hydrogen peroxide. *Biochimica et Biophysica Acta (BBA)-General Subjects* 1380**,** 55-63.

Yamada, Y., Koibuchi, M., Miyamoto, K., Ueda, J., and Uheda, E. (2015). Breakdown of middle lamella pectin by ·OH during rapid abscission in *Azolla*. *Plant, Cell & Environment***.** *In press.* doi: 10.1111/pce.12505.
